# Hypertensive Disorders during Pregnancy and Anthropometric Measurement of Children up to 7 Years of Age: The Hokkaido Birth Cohort Study in Japan

**DOI:** 10.3390/ijerph182010951

**Published:** 2021-10-18

**Authors:** Kritika Poudel, Sumitaka Kobayashi, Chihiro Miyashita, Takeshi Yamaguchi, Naomi Tamura, Atsuko Ikeda-Araki, Yu Ait Bamai, Sachiko Itoh, Keiko Yamazaki, Hideyuki Masuda, Mariko Itoh, Reiko Kishi

**Affiliations:** 1 Center for Environmental and Health Sciences, Hokkaido University, North-12, West-7, Kita-ku, Sapporo 060-0812, Japan; kpoudel@hs.hokudai.ac.jp (K.P.); sukobayashi@cehs.hokudai.ac.jp (S.K.); miyasita@med.hokudai.ac.jp (C.M.); takeshi7698@med.hokudai.ac.jp (T.Y.); ntamura@cehs.hokudai.ac.jp (N.T.); aaraki@cehs.hokudai.ac.jp (A.I.-A.); u-aitbamai@med.hokudai.ac.jp (Y.A.B.); vzbghjn@den.hokudai.ac.jp (S.I.); kyamazaki@cehs.hokudai.ac.jp (K.Y.); hmasuda@cehs.hokudai.ac.jp (H.M.); mitoh@cehs.hokudai.ac.jp (M.I.); 2 Faculty of Health Sciences, Hokkaido University, North 12, West-5, Kita-ku, Sapporo 060-0812, Japan

**Keywords:** hypertensive disorders during pregnancy, anthropometric measurement, child, Hokkaido study, cohort, weight, height, sex-difference, pregnancy, Japan

## Abstract

Hypertensive disorders during pregnancy (HDP) increase the risk of offspring with a low birth weight, preterm birth and small-for-gestational age; however, evidence of the anthropometric measurements during early childhood remains limited. We aimed to understand the associations between maternal HDP and anthropometric measurements of children aged up to seven years in a Japanese cohort. In total, 20,926 mother–infant pairs participated in the Hokkaido Study on Environment and Children’s Health, Japan, from 2002 to 2013. Medical reports were used to confirm HDP exposure, while weight, height, height z score, and weight z score were the outcomes. The prevalence of HDP in the study population was 1.7%. The birth height of male children born to HDP mothers was smaller as compared to those born to non-HDP mothers. When adjusted with covariates, the linear regressions showed significant changes in birth weight (β: −79.3; 95% confidence interval [CI]: −154.8, −3.8), birth height (−0.67; 95% CI: −1.07, −0.26), weight at seven years (1.21; 95% CI: 0.13, 2.29), and weight gain between four and seven years (1.12; 95% CI: 0.28, 1.96) of male children exposed to HDP. Differences were more significant in male children than female. Our study showed that despite low birth weight, male children exposed to HDP caught up with their growth and gained more weight by seven years of age compared with male children not exposed to HDP, whereas no such differences were observed in female children; however, this finding requires replication.

## 1. Introduction

Hypertensive disorders during pregnancy (HDP) are defined as hypertension (blood pressure ≥140/90 mmHg) with or without proteinuria (≥300 mg/24 h), emerging after 20 weeks of gestation. HDP has been classified into four types: preeclampsia, gestational hypertension, superimposed preeclampsia, and chronic hypertension, excluding eclampsia in the previous disease type classification [1,2]. Several studies have shown the impact of HDP on birth outcomes, such as preterm birth and small-for-gestational age, and these infants have a higher need for intensive care units [3,4,5,6]. HDP, including preeclampsia and the hemolytic anemia, elevated liver enzymes, and low platelet count (HELLP) syndrome, increase the risk and severity of fetal growth restriction even after birth [7,8]. HDP is associated with a low birth weight, which is reported to predispose to central obesity, including high body mass index (BMI) combined with a high waist-to-hip ratio, hypertension, and coronary heart disease due to fetal programming [9,10].

The Developmental Origins of Health and Disease (DOHaD) hypothesis may be appropriate for children’s long-term development. Barker’s hypothesis, along with the DOHaD hypothesis, have explained the patterns of the life course in detail, and state that poor fetal nutrition, indicated by small birth size, might lead to fetal adaptations altering the propensity for diseases in adulthood [11,12]. Therefore, it is crucial to describe the growth patterns during childhood to confirm these findings.

Catch-up growth refers to the “a height velocity above the statistical limits of normality for age or maturity during a defined period, following a transient period of growth inhibition; the effect of catch-up growth is to take the child towards his/her pre-retardation growth curve” [13]. Catch-up growth also occurs in regard to other growth parameters such as body weight, body composition, head circumference, sitting height, and leg length [14]. The most remarkable difference in rates of weight gain is seen in the first one to two years of life when infants might show significant “catch up” or “catch down” in their weight [15]. Generally, BMI, which is used as an index of obesity, increases during the first year of life, decreases subsequently, and reaches a nadir. After this, BMI increases throughout childhood, and this second rise is called adiposity rebound [16]. Birth weight is the measure of fetal growth [17]. A high BMI at the age of five and seven years has been suggested to be associated with obesity in adulthood [18]. A substantial increase in adiposity as compared to height has been associated with a higher risk for cardiovascular disease [14]. Early catch-up growth is a risk factor for childhood obesity [19].

Early postnatal growth is strongly affected by genetic factors, and infants genetically predisposed to a large size, but born to small mothers, show fast natal growth [20]. In developed countries, catch-up occurs during the first six months of life and continues during infancy and adolescence. However, in developing countries, the compensatory growth of children born with intrauterine growth restriction (IUGR) has not been observed until six years of age. Therefore, these children remain shorter in height [21]. This suggests that their growth is chronically affected due to unfavorable intrauterine conditions. The intrauterine environment plays an essential role in shaping the children’s growth during their childhood [22]. Different maternal factors, such as smoking, alcohol use, low socioeconomic status, and hypertensive disorders, cause IUGR. Although previous studies have assessed the effect of smoking, alcohol consumption, and low socio-economic status on the prenatal and postnatal health of Japanese children, there is a lack of studies on the association between HDP and early anthropometric growth in children [23,24,25,26]. Moreover, there are limited reports on the association between HDP and anthropometric factors of children up to seven years of age in Japan. Therefore, in this study, we explored growth of children in regard to anthropometric factors, in a cohort of children born to mothers with HDP. We aimed to determine the changes in height, weight, the height for age z score, and the weight for age z score while considering the maternal characteristics.

## 2. Materials and Methods

### 2.1. Participants

**Figure 1:**
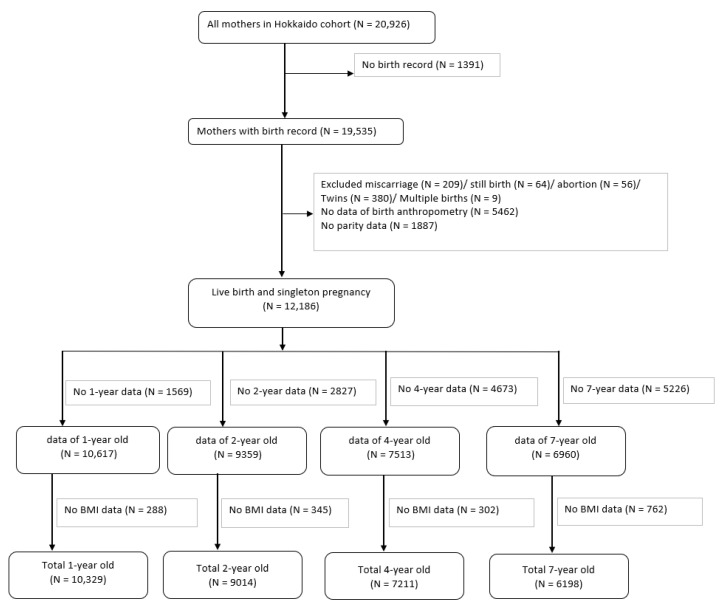
Flowchart of the study.

The Hokkaido Study on Environment and Children’s Health is a prospective birth cohort that was started in 2002; further information about this cohort can be found elsewhere [27,28,29]. From February 2003 to March 2012, the Hokkaido cohort included Japanese women recruited during early pregnancy (13 weeks of gestation), who visited the maternity unit in one of the 37 hospitals and clinics in the Hokkaido Prefecture, Japan. These 37 health services covered the entire Hokkaido area. Figure 1 shows the flowchart of the participants. The cohort consisted of 20,926 pregnant women. As the focus of this study was HDP, we selected mothers who had their medical records of pregnancy. We excluded mothers with miscarriages, stillbirths, abortions, twins, and multiple births. To calculate z-scores, mothers who did not have information on parity and anthropometry of their children at birth were excluded. Thus, we included mothers with live births and singleton pregnancies. Children with no information on anthropometric measurements at one, two, four, and seven years were excluded. We included 12,186 participants from the time of their birth. In total, we included the data obtained from participants at one year (*n* = 10,329), two years (*n* = 9014), four years (*n* = 7211), and seven years (*n* = 6198), and assessed the associations between HDP and anthropometric measurements.

### 2.2. Questionnaire and Medical Records

Participants completed a self-administered questionnaire comprising information on parental characteristics. Baseline data on maternal age, height, weight, education, socioeconomic status, smoking, alcohol consumption before and during pregnancy, and use of artificial reproductive technology were obtained. The medical record provided information on gestational days at delivery, sex, birth height and weight of the infant, and history of HDP of the mother. The exposure variable was HDP. We used the Japanese standard definition of HDP and classified the symptoms based on severity. Mild HDP (h) was defined as a blood pressure ≥140/90 mmHg but <160/110 mmHg after 20 weeks of gestation, and proteinuria (p) ≥300 mg/24 h without exceeding 2.0 g/24 h or a score of 3 + on the dipstick. Severe HDP (H) was defined as a blood pressure of ≥160/110 mmHg and proteinuria (P) exceeding 2.0 g/24 h or a score of 3 + on the dipstick [2].

### 2.3. Anthropometric Measurement

The primary outcomes were weight, height, weight gain, and height gain. The weight and height of each infant measured at birth were defined as birth weight (g) and birth height (cm), respectively. Birth weight, birth height, and z-score were defined as the respective standard deviation (SD) for gestational age in the normal distribution, accounting for infant sex, maternal parity, and gestational age according to the guidelines of the Japan Pediatric Society, created by The Japanese Society for Pediatric Endocrinology [26,30,31].

Height and weight gain were estimated by subtracting the height and weight at birth, respectively, at different age intervals. Anthropometric data at birth were obtained from the birth records. Anthropometric data at one year were obtained after the infant reached one year of age. While the data at two years were obtained from medical checkups during a year and a half of age. Data at four years of age were obtained from the latest checkup at around four years of age. These data were obtained from the Maternal and Child Health Handbook (MCH Handbook) by the parents. The MCH handbook system in Japan was developed to promote the health of all children [32]. The data at seven years of age were obtained from the physical checkup in the first grade at elementary school.

### 2.4. Statistical Analysis

A descriptive analysis was performed on the demographic and socio-personal information of mothers and children over different years. Anthropometric measurements of the children were taken at different ages. Linear regressions were performed to evaluate the association between HDP and height and weight of the infant at different ages. Height gain and weight gain from birth to different ages were compared. The outcomes were adjusted with the following covariates: age at measurement, child’s sex, maternal age, pre-pregnancy BMI, parity, status of smoking during the first trimester, and alcohol consumption during the first trimester. For stratified analysis, sex was excluded from the adjusted covariates. Confidence interval (CI) was set at 95%, with a level of significance at 0.05. All statistical analyses were conducted using IBM SPSS Statistics for Windows (version 27.0; IBM Corp., Armonk, NY, USA).

## 3. Results

### 3.1. Demographic Information

The characteristics of children at different ages are presented in Table 1. Of the 12,186 mothers, 209 (1.7%) mothers had a history of HDP. At baseline, there was no difference in maternal characteristics such as age, BMI, education level, socioeconomic status, smoking, drinking history before and during the first trimester, plasma cotinine levels, in vitro fertilization in the HDP-exposed and non-HDP groups. Similarly, there was no difference in smoking and alcohol consumption habits between HDP-exposed and non-HDP mothers when their children were aged one year, two years, four years, and seven years (data not shown). There was no significant difference between sex, preterm birth, small for gestational age, and birth weight and height among children born from HDP mothers compared to those born to mothers without HDP. There were no differences in height, weight, BMI, height-z-score, weight-z-score, and BMI z-score at one year, two years, four years, and seven years of age in children born to mothers with and without HDP.

The characteristics of male children at different ages are presented in Table 2. There was significant difference in birth height and birth height z-score of male children born to HDP mothers when compared to non HDP mothers. Similarly, when compared at age seven, there was a significant increase in the weight, weight z score and BMI of male children born to HDP mothers.

Similarly, Table 3 presents demographic characteristics of female children. No such difference was observed in the anthropometric measurements from birth to seven years when female children born to non HDP and HDP mothers were compared.

### 3.2. HDP and Anthropometric Measurements

#### 3.2.1. HDP and Weight during Different Years of Age

Table 4 shows the regression analysis of the association between HDP and weight during different years of age. When adjusted for the covariates, compared to male children born to non-HDP mothers, those born to HDP mothers were lighter at birth (mean decrease of −79.3 g [95% CI −154.8, −3.8]). Compared to male children born to non-HDP mothers, those born to HDP mothers were heavier at seven years of age (mean increase of 1.21 kg [95% CI 0.13, 2.29]) after adjusting for the covariates. The weight-for-age z-score had similar findings.

#### 3.2.2. HDP and Height during Different Years of Age

Table 5 shows the association between HDP and height during different years of age. There was a difference in the mean height at birth of male children born to HDP mothers as compared to those born to non-HDP mothers in the crude model (mean decrease, −0.55 cm [95% CI −0.94 −0.17]). Compared to male children born to non-HDP mothers, those born to HDP mothers were shorter at birth (mean −0.67 cm [95% CI −1.07, −0.26]) after adjusting for the covariates. The height for age z-score at birth was similar.

#### 3.2.3. HDP and Difference in Weight and Height Gain at Different Years of Age

Table 6 shows the weight and height gain from birth to different years of age and at different age intervals. Compared to male children born to non- HDP mothers, those born to HDP mothers showed increased weight gain from birth to seven years (mean increase of 1.17 kg [95% CI 0.12, 2.21]) in the crude model and an increase of 1.12 kg ([95% CI 0.16, 2.30]) when adjusted for the covariates. In particular, there was a difference in weight gain at four to seven years of age among male children born to HDP mothers (mean increase of 1.04 kg [95% CI 0.20, 1.87]) in the crude model and a mean increase 1.12 kg [95% CI 0.28, 1.96] when adjusted for covariates. Compared to the children born to non-HDP mothers, the children born to HDP mothers had shorter height gain (mean decrease −1.17 [95% CI −2.16, −0.18]). The difference was marginal when stratified by sex.

Some changes were observed when the BMI of the children was compared at different years of age. Compared to male children born to non-HDP mothers, the children born to HDP mothers had higher BMI at seven years of age (mean increase of 0.57 kg/m^2^ [95% CI 0.04, 1.11]) (Supplementary Table S1). Similarly, compared to male children born to non-HDP mothers, the children born to HDP mothers had a higher BMI gain from birth to seven years, when adjustments with covariates were made (0.61 [95% CI 0.01, 1.22]) (Supplementary Table S2). No other association was observed.

## 4. Discussion

In the present population-based cohort study, we attempted to understand the association between HDP and anthropometric measurements of children from birth to seven years of age. Intrauterine exposure to HDP was negatively associated with height during birth in male children (*p* < 0.05) while no association was observed in females (*p* < 0.1). HDP can cause adverse effects, resulting in several maternal and neonatal complications leading to fetal distress, preterm birth, stillbirth, low birth weight, and IUGR as suggested by our previous as well as other different studies [5,6,33].

The prevalence of HDP in our study was 1.7%, which is less than that reported in the Japan Environment and Children’s study (3.1%) [34]. The difference might be due to variations in the study group and in the Japanese population of pregnant women. The Japanese national survey showed that the mean height and weight of boys were 49.6 cm and 3.2 kg at birth, 81.3 cm and 10.7 kg at a year and a half of age, and 93.3 cm and 13.8 kg at three years of age, respectively [35]. These findings were consistent with our findings and those of another Japanese study that assessed rapid weight gain during early childhood with cardiovascular risk factors in adolescents [36].

Our study did not show any significant difference in the birth weight of children, regardless of HDP. This might be owing to the mild effect of HDP. Patients with non-severe HDP generally have a favorable prognosis, and most studies report that birth weight, incidence of fetal growth restriction, and preterm birth are similar to those of normal pregnancies [37,38,39,40]. The height, height z-score, weight, and weight z-score during birth were significantly lower among children born to HDP mothers; however, no such difference was observed when comparing children at one, two, and four years of age. At seven years of age, the weight and BMI of male children born to HDP mothers were significantly higher than those of children born from normotensive mothers. The weight gain from four to seven years was higher among children born to mothers with HDP. This might suggest that although the weight of such infants at birth is low, these children catch up with the growth and gain more weight by seven years, as compared to other groups. A study conducted among 6343 participants from the ALSPAC cohort showed that nine-year-old children exposed to gestational hypertension presented with higher BMI and waist circumference than those born to normotensive pregnancies [41,42]. The tracking of weight and growth patterns, especially in children with predisposing factors (HDP mothers and small for gestational age babies) may help in identifying children at increased risk of obesity along with enabling prompt early intervention, even before being overweight is evident. Furthermore, in this study, it is very interesting to note that even if children born from an HDP mother weighed within a normal range at birth, the weight gain was still significantly greater with accelerated catch-up, which might result in the risk of obesity and noncommunicable diseases in the future. A retrospective study conducted among 51,505 children showed that among the adolescents who were obese, the greatest acceleration in annual BMI increments occurred between two and six years of age, with a further increase in BMI percentile thereafter [43].

Our study did not show any association between HDP and anthropometric measurements at different ages in female children. From birth till adolescence, linear growth, weight and BMI trajectories differed between the sexes according to the severity of exposure to HDP. In general, HDP and preeclampsia are negatively associated with linear growth. A cohort study from Norway showed that differences in anthropometric measurements were limited to male children [44]. Another international study conducted in Brazil, Great Britain, Hong Kong, Netherlands, Singapore, and the United States showed that the changes in BMI during childhood and adolescence differ between boys and girls, resulting in the development of age- and sex-specific reference data [45,46]. An Israeli study showed that exposure to preeclampsia was positively associated with weight and BMI of 17 years in boys, but no such association was observed in girls [47]. The discrepancies across studies might be due to differences in race and age at assessment.

Male children exposed to HDP had a lower weight at birth. The vast majority of low-birth-weight infants will show postnatal catch-up growth, and most of this catch-up occurred in the first 6–12 months of life, regardless of the height or weight at birth [19,48,49]. Male children born to HDP mothers had a positive weight gain from four to seven years of age. The height gain of the male children from HDP mothers was marginally associated from birth to one year, birth to two years, and one to two years of age. This finding is supported by another cohort study which showed that exposure to preeclampsia was associated with accelerated height gain during early childhood [50]. Another cohort study showed that children exposed to preeclampsia had low birth weight, however, had accelerated catch-up growth postnatally [49]. The effects of preeclampsia on weight trajectories in boys could be influenced by some of the same mechanisms as those for linear growth, such as low levels of IGF-1, mediated through prematurity and inflammation [51]. In our study, there was no difference in height and weight during childhood between children born from HDP and normotensive mothers. In regard to children born from normotensive mothers, similar findings were obtained on the postnatal growth of normal children until 1.5 years of age, excluding preterm and small for gestational age [8]. Children born to HDP mothers were growth-restricted at birth and had complete catch-up growth at 4.5 years of age; however, they were smaller and lighter compared to their peers [7]. Prenatal starvation is associated with epigenetic changes that can persist throughout one’s life, causing a tendency for energy conservation and rapid weight gain [52]. This might explain one of the reasons behind the weight gain of HDP-exposed children from four to seven years.

A Japanese study also showed multiple developmental patterns in boys and girls, suggesting that maternal BMI and some unfavorable behaviors during early pregnancy impacts a child’s pattern of body mass development [53]. Although there are inconsistencies in the literature regarding sex differences in growth after exposure to HDP, boys are more prone to neonatal complications; whether born to term and of adequate birth weight; small for gestational age, and extreme prematurity affects boys more severely than girls [54,55,56]. Our study showed no difference in height, height z-score, weight, and weight z-score of children born to HDP and non-HDP mothers during early childhood. However, this growth pattern could be linked at a low birth weight and the risk of future obesity, suggesting the need for further studies. Studies related to the exposure of HDP and the risk of neurodevelopmental disorders could help understand the casual intrauterine effects. Further research is necessary to understand the health consequences of HDP on the offspring and to observe the transgenerational effect [57].

The strength of this study includes its prospective birth cohort design, comprising 20,926 mother–infant pairs. Owing to the nature of its cohort, we were able to follow up the participants until different years of age. We obtained information on HDP from medical records, which is a highly reliable source. Anthropometric measurements were obtained from the MCH handbook, which is one of the essential records for tracing the health of Japanese children. However, this study has some limitations. The prevalence of HDP was 1.7% in the total participants, which might have led to an underestimation of the results. The data on pre-pregnancy BMI, smoking, and alcohol consumption habits were self-reported by the participants, which might have caused recall biases. Although paternal obesity is also known to accelerate growth in children, we did not have paternal BMI data to compare. Preterm infants with maternal hypertension may have worse outcomes than full-term infants, which could not be assessed in our study due to fewer preterm infants, adding to other limitations. Additionally, the risk of gestational diabetes, type 2 diabetes mellitus, thyroid disorders, and dietary factors were not assessed in this study.

## 5. Conclusions

Our study showed that despite lower weight at birth, male children exposed to HDP had accelerated catch-up growth and gained more weight by seven years as compared to children born to mothers without HDP; however, this finding needs replication. HDP can cause adverse birth outcomes. Hence, identifying risk factors and assessing risk during pregnancy, along with monitoring medical history and proper health education by medical personnel, and public health community interventions can reduce the complications associated with pregnancy. As intrauterine exposure to hypertensive disorders can affect the neurodevelopmental and cardiometabolic health of children in the long run, our study recommends further studies on Japanese mothers to strengthen the evidence provided by our findings.

## Figures and Tables

**Table 1:** Demographic information of children from birth to seven years.

Characteristics	Total (12186)	Non HDP (11977)	HDP (209)	*p*
**Infants at Birth**				
Sex				
Male	6086 (49.9)	5975 (49.9)	111 (53.1)	0.356
Female	6100 (50.1)	6002 (50.1)	98 (46.9)	
Weight at birth (g)	3054.85 ± 387.54	3054.93 ± 387.58	3050.12 ± 386.51	0.859
Height at birth (cm)	49.01 ± 2.08	49.02 ± 2.08	48.86 ± 1.98	0.288
Birth weight z score	0.15 ± 1.01	0.15 ± 1.01	0.13 ± 0.93	0.727
Birth height z score	0.10 ± 0.99	0.10 ± 0.99	0.00 ± 0.86	0.142
**1 year**	**Total (10329)**	**Non HDP (10144)**	**HDP (185)**	***p***
Sex				
Male	5155 (49.9)	5058 (49.9)	97 (52.4)	0.488
Female	5174 (50.1)	5086 (50.1)	88 (47.6)	
Age at measurement (years)	1.13 ± 0.11	1.14 ± 0.11	1.13 ± 1.13	0.629
Height at 1 year (cm)	69.83 ± 6.72	69.82 ± 6.73	70.47 ± 6.07	0.191
Weight at 1 year (kg)	8.35 ± 1.80	8.35 ± 1.80	8.47 ± 1.72	0.363
BMI at 1 year (kg/m^2^)	16.85 ± 1.72	16.85 ± 1.72	16.83 ± 1.61	0.877
Height z score (1 years)	−2.11 ± 2.61	−2.11 ± 2.61	−1.85 ± 2.38	0.173
Weight z score (1 years)	0.0 ± 1.0	−0.00 ± 1.00	0.07 ± 0.95	0.362
BMI z score	0.39 ± 1.28	0.39 ± 1.28	0.37 ± 1.21	0.792
**2 year**	**Total (9014)**	**Non HDP (8848)**	**HDP (166)**	***p***
Sex				
Male	4544 (50.4)	4456 (50.4)	88 (53.0)	0.499
Female	4470 (49.6)	4392 (49.6)	78 (47.0)	
Age at measurement (years)	1.60 ± 0.17	1.60 ± 0.17	1.61 ± 0.17	0.337
Weight at 2 years (kg)	10.56 ± 2.02	10.56 ± 2.03	10.57 ± 1.24	0.943
Height at 2 years (cm)	80.28 ± 2.86	80.28 ± 2.85	80.48 ± 3.01	0.380
BMI at 2 year (kg/m^2^)	16.36 ± 3.31	16.36 ± 3.33	16.28 ± 1.30	0.75
Height z score (2 years)	−0.07 ± 1.12	−0.07 ± 1.12	−0.06 ± 1.16	0.88
Weight z score (2 years)	0.00 ± 1.00	−0.00 ± 1.00	0.00 ± 0.61	0.943
BMI z score	0.33 ± 1.17	0.33 ± 1.17	0.30 ± 1.01	0.734
**4 year**	**Total (7211)**	**Non HDP (7066)**	**HDP (145)**	***p***
Sex				
Male	3654 (50.7)	3581 (50.7)	73 (50.3)	0.936
Female	3557 (49.3)	3485 (49.3)	72 (49.7)	
Age at measurement (years)	3.15 ± 0.69	3.15 ± 0.70	3.11 ± 0.34	0.488
Weight at 4 years (kg)	13.82 ± 1.55	13.82 ± 1.54	13.77 ± 1.65	0.664
Height at 4 years (cm)	93.20 ± 3.57	93.20 ± 3.57	93.26 ± 3.44	0.845
BMI at 4 year (kg/m^2^)	15.88 ± 1.28	15.89 ± 1.28	15.79 ± 1.19	0.350
Height z score (4 yrs)	−0.09 ± 1.07	−0.09 ± 1.07	−0.05 ± 0.95	0.631
Weight z score (4 yrs)	0.00 ± 1.00	0.00 ± 1.00	−0.04 ± 1.07	0.664
BMI z score	0.35 ± 0.92	0.35 ± 0.91	0.27 ± 0.93	0.307
**7 year**	**Total (6198)**	**Non HDP (6085)**	**HDP (118)**	***p***
Sex				
Male	3140 (50.7)	3080 (50.6)	60 (50.8)	0.967
Female	3058 (49.3)	3000 (49.4)	58 (49.2)	
Age at measurement (years)	7.01 ± 0.32	7.01 ± 0.32	7.01 ± 0.25	0.960
Weight at 7 years (kg)	22.68 ± 3.95	22.67 ± 3.94	23.11 ± 4.50	0.227
Height at 7 years (cm)	119.73 ± 5.39	119.73 ± 5.39	119.74 ± 5.39	0.978
BMI at 7 years (kg/m^2^)	15.75 ± 2.00	15.75 ± 1.99	16.02 ± 2.24	0.146
Weight z score (7 years)	−0.00 ± 0.90	−0.01 ± 0.90	0.09 ± 1.02	0.227
Height z score (7 years)	0.15 ± 1.05	0.15 ± 1.05	0.15 ± 1.04	0.984
BMI z score	−1.09 ± 1.02	−0.11 ± 1.02	0.01 ± 1.09	0.213

Chi-squared test for categorical data and *t*-test for continuous data; *p*-values are the statistical tests between non-HDP and HDP groups.

**Table 2:** Demographic information of male children from birth to seven years.

Characteristics of Male	Total (6086)	Non HDP (5975)	HDP (111)	*p*
**Infants at Birth**				
Weight at birth (g)	3096.46 ± 386.15	3097.57 ± 385.44	3036.34 ± 420.26	0.098
Height at birth (cm)	49.28 ± 2.06	49.29 ± 2.06	48.73 ± 2.23	0.005
Birth weight z score	0.15 ± 1.02	0.15 ± 1.02	0.02 ± 0.93	0.198
Birth height z score	0.27 ± 0.97	0.27 ± 0.97	0.02 ± 0.92	0.005
**1 year**	**Total (5155)**	**Non HDP (5058)**	**HDP (97)**	***p***
Age at measurement (years)	1.14 ± 0.11	1.14 ± 0.11	1.14 ± 0.12	0.532
Height at 1 year (cm)	70.52 ± 6.73	70.51 ± 6.73	71.27 ± 6.31	0.268
Weight at 1 year (kg)	8.61 ± 1.83	8.61 ± 1.83	8.73 ± 1.78	0.537
BMI at 1 year (kg/m^2^)	17.05 ± 1.73	17.05 ± 1.73	16.95 ± 1.69	0.602
Height z score (1 years)	−2.10 ± 2.59	−2.11 ± 2.59	−1.84 ± 2.43	0.311
Weight z score (1 years)	0.14 ± 1.01	−0.14 ± 1.01	0.21 ± 0.99	0.537
BMI z score	0.36 ± 1.28	0.36 ± 1.27	0.30 ± 1.23	0.644
**2 year**	**Total (4544)**	**Non HDP (4456)**	**HDP (88)**	***p***
Age at measurement (years)	1.59 ± 0.17	1.60 ± 0.17	1.59 ± 0.10	0.993
Weight at 2 years (kg)	10.81 ± 1.51	10.81 ± 1.51	10.88 ± 1.26	0.671
Height at 2 years (cm)	80.89 ± 2.87	80.89 ± 2.87	81.05 ± 3.06	0.584
BMI at 2 year (kg/m^2^)	16.51 ± 2.40	16.51 ± 2.41	16.53 ± 1.29	0.939
Height z score (2 years)	−0.06 ± 1.11	−0.06 ± 1.12	−0.01 ± 1.02	0.687
Weight z score (2 years)	0.13 ± 0.75	0.13 ± 0.75	0.16 ± 0.62	0.671
BMI z score	0.30 ± 0.98	0.30 ± 0.98	0.34 ± 1.04	0.733
**4 year**	**Total (3654)**	**Non HDP (3581)**	**HDP (73)**	***p***
Age at measurement (years)	3.15 ± 0.24	3.15 ± 0.24	3.10 ± 0.18	0.080
Weight at 4 years (kg)	14.04 ± 1.53	14.04 ± 1.52	14.03 ± 1.74	0.952
Height at 4 years (cm)	93.72 ± 3.44	93.72 ± 3.44	93.65 ± 3.28	0.855
BMI at 4 year (kg/m^2^)	15.96 ± 1.17	15.96 ± 1.17	15.96 ± 1.28	0.978
Height z score (4 years)	−0.11 ± 1.03	−0.11 ± 1.02	−0.03 ± 0.92	0.510
Weight z score (4 years)	0.14 ± 0.99	0.14 ± 0.98	0.14 ± 1.13	0.952
BMI z score	0.37 ± 0.92	0.37 ± 0.92	0.35 ± 1.02	0.820
**7 year**	**Total (3140)**	**Non HDP (3080)**	**HDP (60)**	***p***
Age at measurement (years)	7.01 ± 0.32	7.01 ± 0.32	7.03 ± 0.31	0.604
Weight at 7 years (kg)	23.02 ± 4.16	23.00 ± 4.13	24.171 ± 5.09	0.031
Height at 7 years (cm)	120.01 ± 5.39	120.00 ± 5.39	120.65 ± 4.84	0.346
BMI at 7 years (kg/m^2^)	15.91 ± 2.09	15.90 ± 2.09	16.47 ± 2.54	0.036
Weight z score (7 years)	0.07 ± 0.94	0.07 ± 0.94	0.33 ± 1.15	0.031
Height z score (7 years)	0.12 ± 1.04	0.12 ± 1.05	0.23 ± 0.97	0.427
BMI z score	−0.05 ± 1.06	−0.06 ± 1.06	0.18 ± 1.17	0.081

Chi-squared test for categorical data and *t*-test for continuous data; *p*-values are the statistical tests between non-HDP and HDP groups.

**Table 3:** Demographic information on female children from birth to seven years.

Characteristics of Female	Total (6100)	Non HDP (6002)	HDP (98)	*p*
**Infants at Birth**				
Weight at birth (g)	3013.27 ± 384.59	3012.41 ± 385.16	3065.72 ± 345.82	0.173
Height at birth (cm)	48.75 ± 2.06	48.74 ± 2.07	49.01 ± 1.65	0.212
Birth weight z score	0.15 ± 1.00	0.15 ± 1.00	0.24 ± 0.93	0.377
Birth height z score	−0.06 ± 0.99	−0.06 ± 0.99	−0.01 ± 0.79	0.595
**1 year**	**Total (5174)**	**Non HDP (5086)**	**HDP (88)**	***p***
Age at measurement (years)	1.13 ± 0.11	1.14 ± 0.11	1.12 ± 0.13	0.178
Height at 1 year (cm)	69.14 ± 6.63	69.13 ± 6.65	69.59 ± 5.69	0.526
Weight at 1 year (kg)	8.10 ± 1.74	8.09 ± 1.75	8.19 ± 1.62	0.591
BMI at 1 year (kg/m^2^)	16.65 ± 1.70	16.85 ± 1.70	16.69 ± 1.53	0.831
Height z score (1 years)	−2.11 ± 2.63	−2.11 ± 2.63	−1.85 ± 2.33	0.361
Weight z score (1 years)	−0.13 ± 0.97	−0.14 ± 0.97	−0.09 ± 0.89	0.591
BMI z score	0.43 ± 1.28	0.43 ± 1.29	0.44 ± 1.20	0.899
**2 year**	**Total (4470)**	**Non HDP (4392)**	**HDP (78)**	***p***
Age at measurement (years)	1.60 ± 0.18	1.60 ± 0.18	1.62 ± 0.23	0.176
Weight at 2 years (kg)	10.30 ± 2.40	10.30 ± 2.42	10.22 ± 1.12	0.763
Height at 2 years (cm)	79.67 ± 2.71	79.67 ± 2.71	79.82 ± 2.84	0.609
BMI at 2 year (kg/m^2^)	16.21 ± 4.02	16.22 ± 4.05	16.00 ± 1.25	0.641
Height z score (2 years)	−0.08 ± 1.13	−0.09 ± 1.12	−0.11 ± 1.30	0.831
Weight z score (2 years)	−0.13 ± 1.12	−0.13 ± 1.20	−0.17 ± 0.55	0.763
BMI z score	0.36 ± 1.33	0.37 ± 1.33	0.26 ± 0.99	0.496
**4 year**	**Total (3557)**	**Non HDP (3485)**	**HDP (72)**	***p***
Age at measurement (years)	3.14 ± 0.24	3.14 ± 0.24	3.16 ± 0.24	0.579
Weight at 4 years (kg)	13.59 ± 1.54	13.60 ± 1.54	13.50 ± 1.51	0.586
Height at 4 years (cm)	92.67 ± 3.63	92.66 ± 3.63	92.86 ± 3.58	0.643
BMI at 4 year (kg/m^2^)	15.81 ± 1.37	15.81 ± 1.38	15.61 ± 1.08	0.228
Height z score (4 years)	−0.08 ± 1.11	−0.08 ± 1.11	−0.07 ± 0.98	0.967
Weight z score (4 years)	−0.15 ± 0.99	−0.15 ± 0.99	−0.21 ± 0.98	0.586
BMI z score	0.32 ± 0.91	0.32 ± 0.92	0.19 ± 0.83	0.223
**7 year**	**Total (3058)**	**Non HDP (3000)**	**HDP (58)**	***p***
Age at measurement (years)	7.01 ± 0.32	7.01 ± 0.32	6.99 ± 0.18	0.548
Weight at 7 years (kg)	22.32 ± 3.70	22.33 ± 3.71	22.02 ± 3.52	0.533
Height at 7 years (cm)	119.44 ± 5.41	119.45 ± 5.40	118.79 ± 5.81	0.360
BMI at 7 years (kg/m^2^)	15.59 ± 1.88	15.59 ± 1.88	15.55 ± 1.78	0.858
Weight z score (7 years)	−0.08 ± 0.84	−0.08 ± 0.84	−0.15 ± 0.80	0.533
Height z score (7 years)	0.17 ± 1.06	0.18 ± 1.06	0.07 ± 1.11	0.443
BMI z score	−0.16 ± 0.98	−0.16 ± 0.98	−0.17 ± 0.97	0.941

Chi-squared test for categorical data and *t*-test for continuous data; *p*-values are the statistical tests between non-HDP and HDP groups.

**Table 4:** Association between hypertensive disorders during pregnancy and weight during different periods of age using the multiple linear regression models.

			All		Male		Female	
			Crude	Adjusted	Crude	Adjusted	Crude	Adjusted
Year	Outcome	Exposure	β (95% CI)		β (95% CI)		β (95% CI)	
Birth	Weight (g)	Non-HDP	Reference		Reference		Reference	
		HDP	−4.8 (−57.8, 48.2)	−5.89 (−60.0, 48.3)	−61.23 (−133.7, 11.3) ^†^	−79.3 (−154.8, −3.8) *	53.31 (−23.46, 130.10)	73.8 (−3.9, 151.4) ^†^
1 year	Weight (kg)	Non-HDP	Reference		Reference		Reference	
		HDP	122 (−140.8, 384.7)	78 (−192.6, 348.5)	115.6 (−251.9, 483.1)	45.26 (−343.5, 434.1)	101.06 (−267.2, 469.3)	112.7 (−263.7, 489.1)
2 years	Weight (kg)	Non-HDP	Reference		Reference		Reference	
		HDP	0.01 (−0.30, 0.32)	0.00 (−0.32, 0.33)	0.07 (−0.25, 0.39)	0.07 (−0.27, 0.41)	−0.08 (−0.62, 0.46)	−0.09 (−0.65, 0.47)
4 years	Weight (kg)	Non-HDP	Reference		Reference		Reference	
		HDP	−0.06 (−0.31, 0.20)	−0.02 (−0.28, 0.24)	−0.01 (−0.26, 0.34)	0.05 (−0.32, 0.42)	−0.10 (−0.46, 0.26)	−0.08 (−0.44, 0.28)
7 years	Weight (kg)	Non-HDP	Reference		Reference		Reference	
		HDP	0.44 (−0.28, 1.16)	0.48 (−0.24, 1.20)	1.17 (0.10, 2.23) *	1.21 (0.13, 2.29) *	−0.31 (−1.27, 0.66)	−0.22 (−1.16, 0.72)
Birth	Weight z score	Non-HDP	Reference		Reference		Reference	
		HDP	−0.02 (−0.16, 0.11)	−0.05 (−0.19, 0.08)	−0.13 (−0.32, 0.07)	−0.20 (−0.39, −0.01) *	0.09 (−0.11, 0.29)	0.10 (−0.09, 0.30)
1 year	Weight z score	Non-HDP	Reference		Reference		Reference	
		HDP	0.07 (−0.08, 0.21)	0.04 (−0.11, 0.19)	0.06 (−0.14, 0.27)	0.025 (−0.19, 0.24)	0.06 (−0.15, 0.26)	0.06 (−0.15, 0.27)
2 years	Weight z score	Non-HDP	Reference		Reference		Reference	
		HDP	0.01 (−0.15, 0.16)	0.01 (−0.16, 0.17)	0.03 (−0.12, 0.19)	0.04 (−0.13, 0.21)	−0.04 (−0.31, 0.22)	−0.04 (−0.47, 0.06)
4 years	Weight z score	Non-HDP	Reference		Reference		Reference	
		HDP	−0.04 (−0.20, 0.13)	−0.01 (−0.18, 0.15)	−0.01 (−0.24, 0.22)	0.02 (−0.22, 0.25)	−0.06 (−0.30, 0.17)	−0.05 (−0.28, 0.19)
7 years	Weight z score	Non-HDP	Reference		Reference		Reference	
		HDP	0.10 (−0.06, 0.26)	0.10 (−0.06, 0.27)	0.27 (0.02, 0.51) *	0.28 (0.03, 0.53) *	−0.07 (−0.29, 0.15)	−0.06 (−0.28, 0.15)

^†^*p* < 0.1, * *p* < 0.05; Confidence interval 95%, Adjusted covariates: Child age at measurement, child sex, maternal age, maternal pre pregnancy BMI, parity, smoking during first trimester, alcohol during first trimester; Sex was excluded during stratified analysis.

**Table 5:** Association between hypertensive disorders during pregnancy and height during different ages using the multiple linear regression models.

			All		Male		Female	
			Crude	Adjusted	Crude	Adjusted	Crude	Adjusted
Year	Outcome	Exposure	β (95% CI)	β (95% CI)	β (95% CI)	β (95% CI)	β (95% CI)	β (95% CI)
Birth	Height (cm)	Non-HDP	Reference		Reference		Reference	
		HDP	−0.15 (−0.44, 0.13)	−0.18 (−0.47, 0.11)	−0.55 (−0.94, −0.17) **	−0.67 (−1.07, −0.26) ***	0.26 (−0.15, 0.67)	0.34 (−0.08, 0.76)
1 year	Height (cm)	Non-HDP	Reference		Reference		Reference	
		HDP	0.65 (−0.33, 1.63)	0.42 (−0.59, 1.44)	0.76 (−0.59, 2.11)	0.32 (−1.11, 1.75)	0.45 (−0.95, 1.85)	0.53 (−0.90, 1.96)
2 year	Height (cm)	Non-HDP	Reference		Reference		Reference	
		HDP	0.20 (−0.24, 0.64)	0.15 (−0.29, 0.59)	0.17 (−0.43, 0.77)	0.09 (−0.54, 0.72)	0.11 (−0.49, 0.71)	0.20 (−0.41, 0.82)
4 year	Height (cm)	Non-HDP	Reference		Reference		Reference	
		HDP	0.09 (−0.48, 0.67)	0.01 (−0.59, 0.61)	−0.07 (−0.87, 0.72)	−0.16 (−0.99, 0.68)	0.20 (−0.65, 1.05)	0.20 (−0.66, 1.06)
7 year	Height (cm)	Non-HDP	Reference		Reference		Reference	
		HDP	0.01 (−0.97, 1.00)	−0.06 (−1.06, 0.94)	0.66 (−0.71, 2.03)	0.32 (−1.10, 1.75)	−0.66 (−2.06,0.75)	−0.40 (−1.79, 1.00)
Birth	Height z score	Non-HDP	Reference		Reference		Reference	
		HDP	−0.10 (−0.24, 0.03)	−0.12 (−0.26, 0.02) ^†^	−0.26 (−0.44, −0.08) **	−0.31 (−0.50, −0.12) ***	0.05 (−0.14, 0.25)	0.08 (−0.13, 0.28)
1 year	Height z score	Non-HDP	Reference		Reference		Reference	
		HDP	0.26 (−0.11, 0.64)	0.20 (−0.19, 0.60)	0.27 (−0.25, 0.79)	0.14 (−0.41, 0.69)	0.26 (−0.30, 0.81)	0.26 (−0.30, 0.83)
2 year	Height z score	Non-HDP	Reference		Reference		Reference	
		HDP	0.01 (−0.16, 0.19)	0.01 (−0.17, 0.19)	0.05 (−0.19, 0.28)	0.01 (−0.24, 0.26)	−0.03 (−0.28, 0.23)	0.00 (−0.26, 0.26)
4 year	Height z score	Non-HDP	Reference		Reference		Reference	
		HDP	0.04 (−0.13, 0.22)	0.02 (−0.17, 0.20)	0.08 (−0.16, 0.32)	0.02 (−0.23, 0.28)	0.01 (−0.25, 0.26)	0.003 (−0.26,0.27)
7 year	Height z score	Non-HDP	Reference		Reference		Reference	
		HDP	0.002 (−0.19, 0.19)	−0.01 (−0.21, 0.19)	0.11 (−0.16, 0.38)	0.06 (−0.22, 0.35)	−0.11 (−0.38, 0.17)	−0.08 (−0.36, 0.21)

^†^*p* < 0.1, ** *p* < 0.01, *** *p* < 0.001; CI: Confidence interval; Adjusted covariates: Child age at measurement, child sex, maternal age, maternal pre pregnancy BMI, parity, smoking during first trimester, alcohol during first trimester. Sex was excluded from stratified analysis.

**Table 6:** Difference in weight and height gain from birth to different years of ages and at different age intervals using the multiple linear regression models.

			Total		Male		Female	
			Crude	Adjusted	Crude	Adjusted	Crude	Adjusted
Variables	Mean Score	Exposure	β (95% CI)	β (95% CI)	β (95% CI)	β (95% CI)	β (95% CI)	β (95% CI)
**Weight gain**								
Birth–1 year (g)	5298.9 ± 1770.4	Non-HDP	Reference		Reference		Reference	
	5421.1 ± 1713	HDP	122.2(−135.1379.5)	83.2 (−182.9, 349.4)	184.3 (−175.6, 544.2)	136.4 (−245.3, 518.1)	30.5 (−332.2, 393.3)	30.0 (−341.1, 401.1)
Birth–2 year (kg)	7.50 ± 2.0	Non-HDP	Reference		Reference		Reference	
	7.50 ± 1.2	HDP	0.00(−0.30, 0.31)	−0.002 (−0.33, 0.32)	0.10 (−0.21, 0.41)	0.13 (−0.20, 0.45)	−0.13 (−0.67, 0.40)	−0.15 (−0.71, 0.41)
Birth–4 year (kg)	10.77 ± 1.46	Non-HDP	Reference		Reference		Reference	
	10.71 ± 1.58	HDP	−0.06 (−0.31, 0.18)	−0.02 (−0.27, 0.22)	0.03 (−0.31, 0.36)	0.11 (−0.24, 0.46)	−0.16 (−0.50, 0.16)	−0.15 (−0.49, 0.20)
Birth–7 year (kg)	19.62 ± 3.89	Non-HDP	Reference		Reference		Reference	
	20.04 ± 4.40	HDP	0.42 (−0.29, 1.13)	0.46 (−0.25, 1.17)	1.17 (0.12, 2.21) *	1.12 (0.16, 2.30) *	−0.36 (−1.31, 0.59)	−0.29 (−1.22, 0.65)
1 year–2 year (kg)	2.17 ± 2.26 ^†^	Non-HDP	Reference		Reference		Reference	
	1.86 ± 1.21	HDP	−0.31 (−0.66, 0.04) ^†^	−0.28 (−0.65, 0.08)	−0.24 (−0.66, 0.18)	−0.17 (−0.61, 0.27)	−0.39 (−0.96, 0.18)	−0.37 (−0.96, 0.21)
2 year–4 year (kg)	3.27 ± 2.03	Non-HDP	Reference		Reference		Reference	
	3.25 ± 0.79	HDP	−0.02 (−0.35, 0.32)	0..01 (−0.35, 0.37)	−0.05 (−0.25, 0.14)	−0.03 (−0.24, 0.18)	0.02 (−0.64, 0.68)	0.06 (−0.63, 0.75)
4 year–7 year (kg)	8.78 ± 3.68	Non-HDP	Reference		Reference		Reference	
	9.27 ± 3.42	HDP	0.49 (−0.20, 1.18)	0.47 (−0.24, 1.18)	1.04 (0.20, 1.87) *	1.12 (0.28, 1.96) **	−0.10 (−1.23, 1.02)	−0.19 (−1.34, 0.95)
**Height gain**								
Birth–1 year (cm)	20.82 ± 6.72	Non-HDP	Reference		Reference		Reference	
	21.57 ± 6.17	HDP	0.76 (−0.22, 1.73)	0.58 (−0.43, 1.60)	1.32 (−0.03, 2.67) ^†^	1.04 (−0.39, 2.47)	0.09 (−1.31, 1.50)	0.12 (−1.32, 1.56)
Birth–2 year (cm)	31.26 ± 2.79	Non-HDP	Reference		Reference		Reference	
	31.50 ± 2.68	HDP	0.23 (−0.19, 0.66)	0.23 (−0.20, 0.67)	0.52 (−0.07, 1.11) ^†^	0.57 (−0.04, 1.19) ^†^	−0.13 (−0.75, 0.49)	−0.11 (−0.74, 0.51)
Birth–4 year (cm)	44.21 ± 3.50	Non-HDP	Reference		Reference		Reference	
	44.25 ± 3.14	HDP	0.05 (−0.53, 0.62)	0.06 (−0.54, 0.65)	0.27 (−0.52, 1.05)	0.32 (−0.50, 1.15)	−0.17 (−1.01, 0.66)	−0.20 (−1.05, 0.65)
Birth–7 year (cm)	70.78 ± 5.32	Non-HDP	Reference		Reference		Reference	
	70.69 ± 4.81	HDP	−0.09 (−1.06, 0.88)	−0.11 (−1.09, 0.87)	0.82 (−0.53, 2.17)	0.66 (−0.73, 2.06)	−1.02 (−2.41, 0.36)	−0.83 (−2.21, 0.55)
1 year–2 year (cm)	10.33 ± 6.39 *	Non-HDP	Reference		Reference		Reference	
	9.16 ± 4.32	HDP	−1.17 (−2.16, −0.18) *	−0.97 (−2.0, 0.06) ^†^	−1.21 (−2.58, 0.15) ^†^	−0.92 (−2.37, 0.53)	−1.11 (−2.55, 0.34)	−0.99 (−2.45, 0.48)
2 year–4 year (cm)	12.96 ± 2.27	Non-HDP	Reference		Reference		Reference	
	12.71 ± 1.81	HDP	−0.25 (−0.62, 0.13)	−0.28 (−0.68, 0.11)	−0.31 (−0.80, 0.18)	−0.39 (−0.91. 0.13)	−0.18 (−0.76, 0.40)	−0.19 (−0.79, 0.40)
4 year–7 year (cm)	26.38 ± 4.36	Non-HDP	Reference		Reference		Reference	
	26.22 ± 3.03	HDP	−0.16 (−0.98, 0.66)	−0.19 (−1.02, 0.63)	0.22 (−0.99, 1.43)	0.18 (−1.08, 1.45)	−0.56 (−1.66, 0.53)	−0.56 (−1.63, 0.50)

^†^*p* < 0.1, * *p* < 0.05, ** *p* < 0.01, Confidence interval 95%, Adjusted covariates: Child age at measurement, child sex, maternal age, maternal pre pregnancy BMI, parity, smoking during first trimester, alcohol during first trimester; For stratified analysis, sex was excluded from adjusted covariates.

## Data Availability

The data are not publicly available due to ethical restrictions and specific legal framework in Japan. All inquiries should be addressed to Reiko Kishi, principal investigator of the Hokkaido Study on Environment and Children’s Health, Center for Environmental and Health Sciences, Hokkaido University.

## Supplementary Materials

The following are available online at https://www.mdpi.com/article/10.3390/ijerph182010951/s1. Table S1: Association between hypertensive disorders during pregnancy and body mass index during different years of age; Table S2: Association between hypertensive disorders during pregnancy and difference in body mass index gain at different age intervals.
