# Supplementary material for: Hypertensive Disorders during Pregnancy and Anthropometric Measurement of Children up to 7 Years of Age: The Hokkaido Birth Cohort Study in Japan
Source: Int J Environ Res Public Health. 2021 Oct 18;18(20):10951. doi: 10.3390/ijerph182010951 (PMC8536157; doi:10.3390/ijerph182010951)
Supplement: Supplementary file 1 [file ijerph-18-10951-s001.zip › ijerph-1370233-supplementary.pdf]

Table S1. Association between hypertensive disorders during pregnancy and body mass index during different years of ages.

			All		Male		Female	
			Crude	Adjusted	Crude	Adjusted	Crude	Adjusted
**Year**	**Outcome**	**Exposure**	**β (95% CI)**	**β (95% CI)**	**β (95% CI)**	**β (95% CI)**	**β (95% CI)**	**β (95% CI)**
Birth	BMI	Non-HDP	Reference	Reference	Reference	Reference	Reference	Reference
		HDP	0.06 (-0.09, 0.21)	0.06 (-0.09, 0.22)	0.02 (-0.19, 0.22)	-0.004 (-0.22, 0.21)	0.10 (-0.13, 0.32)	0.14 (-0.09, 0.37)
1 year	BMI	Non-HDP	Reference	Reference	Reference	Reference	Reference	Reference
		HDP	-0.02 (-0.27, 0.23)	-0.014 (-0.27, 0.24)	-0.09 (-0.44, 0.25)	-0.04 (-0.40, 0.33)	0.04 (-0.32, 0.40)	0.012 (-0.35, 0.38)
2 year	BMI	Non-HDP	Reference	Reference	Reference	Reference	Reference	Reference
		HDP	-0.08 (-0.59, 0.42)	-0.08 (-0.63, 0.46)	0.02 (-0.47, 0.53)	0.06 (-0.49, 0.60)	-0.21 (-1.11, 0.69)	-0.25 (-1.19, 0.70)
4 year	BMI	Non-HDP	Reference	Reference	Reference	Reference	Reference	Reference
		HDP	-0.10 (-0.31, 0.11)	-0.04 (-0.26, 0.17)	-0.003 (-0.27, 0.27)	0.09 (-0.19, 0.37)	-0.20 (-0.52, 0.12)	-0.17 (-0.49, 0.16)
7 year	BMI	Non-HDP	Reference	Reference	Reference	Reference	Reference	Reference
		HDP	0.27 (-0.09, 0.63)	0.32 (-0.04, 0.69)†	0.57 (0.04, 1.11)*	0.56 (0.03, 1.10)*	-0.05 (-0.53, 0.44)	-0.04 (-0.53, 0.45)
Birth	BMI z score	Non-HDP	Reference	Reference	Reference	Reference	Reference	Reference
		HDP	0.05 (-0.08, 0.19)	0.06 (-0.08, 0.20)	0.01 (-0.17, 0.20)	-0.004 (-0.20, 0.19)	0.09 (-0.11, 0.29)	0.13 (-0.96, -0.50)
1 year	BMI z score	Non-HDP	Reference	Reference	Reference	Reference	Reference	Reference
		HDP	-0.02 (-0.21, 0.16)	-0.013 (-0.21, 0.18)	-0.6 (-0.32, 0.20)	-0.03 (-0.30, 0.24)	0.02 (-0.25, 0.29)	0.003 (-0.27, 0.28)
2 year	BMI z score	Non-HDP	Reference	Reference	Reference	Reference	Reference	Reference
		HDP	-0.03 (-0.21, 0.15)	-0.012 (-0.20, 0.18)	0.04 (-0.17, 0.24)	0.07 (-0.14, 0.29)	-0.10 (-0.40, 0.19)	-0.11 (-0.42, 0.19)
4 year	BMI z score	Non-HDP	Reference	Reference	Reference	Reference	Reference	Reference
		HDP	-0.08 (-0.23, 0.07)	-0.03 (-0.18, 0.12)	-0.02 (-0.24, 0.19)	0.06 (-0.16, 0.28)	-0.13 (-0.35, 0.08)	-0.11 (-0.33, 0.10)
7 year	BMI z score	Non-HDP	Reference	Reference	Reference	Reference	Reference	Reference
		HDP	0.12 (-0.07, 0.30)	0.15 (-0.04, 0.34)	0.24 (-0.03, 0.51)†	0.31 (0.03, 0.60)*	-0.01 (-0.26, 0.25)	-0.02 (-0.27, 0.23)

BMI was measured in kg/m^2. †p<0.1, * p<0.05; Confidence interval 95%, Adjusted covariates: Age at measurement, mothers age, mothers pre pregnancy BMI, parity, smoking during first trimester, alcohol during first trimester. Sex was excluded from stratified analysis.

Table S2. Association between hypertensive disorders during pregnancy and difference in body mass index gain at different age intervals.

			Total		Male		Female	
			**Crude**	**Adjusted**	**Crude**	**Adjusted**	**Crude**	**Adjusted**
**BMI gain**	**Mean score**	**Exposure**	**β (95% CI)**	**β (95% CI)**	**β (95% CI)**	**β (95% CI)**	**β (95% CI)**	**β (95% CI)**
Birth-1 yr	4.17±1.87	Non- HDP	Reference	Reference	Reference	Reference	Reference	Reference
	4.10±1.82	HDP	-0.07 (-0.34, 0.20)	-0.07 (-0.35, 0.21)	-0.07 (-0.45, 0.30)	-0.01 (-0.40, 0.39)	-0.09 (-0.48, 0.31)	-0.13 (-0.54, 0.27)
Birth-2 yr	3.69±3.45	Non- HDP	Reference	Reference	Reference	Reference	Reference	Reference
	3.55±1.51	HDP	-0.14 (-0.66, 0.39)	-0.15 (-0.71, 0.41)	-0.01 (-0.55, 0.53)	0.40 (-0.54, 0.62)	-0.29 (-1.1, 0.63)	-0.36 (-1.32, 0.61)
Birth- 4 yr	3.22±1.53	Non- HDP	Reference	Reference	Reference	Reference	Reference	Reference
	3.09±1.49	HDP	-0.13 (-0.38, 0.16)	-0.09 (-0.35, 0.17)	-0.01 (-0.34, 0.32)	0.10 (-0.25, 0.45)	-0.26 (-0.64, 0.12)	-0.26 (-0.65, 0.12)
Birth-7 yr	3.07±2.18	Non- HDP	Reference	Reference	Reference	Reference	Reference	Reference
	3.27±2.28	HDP	0.20 (-0.20, 0.60)	0.24 (-0.17, 0.65)	0.48 (-0.10, 1.01)	0.61 (0.01, 1.22)*	-0.09 (-0.63, 0.45)	-0.12 (-0.67, 0.43)
1 yr-2 yr	-0.51±3.29	Non- HDP	Reference	Reference	Reference	Reference	Reference	Reference
	-0.72±1.06	HDP	-0.21 (-0.72, 0.30)	-0.25 (-0.79, 0.28)	-0.03 (-0.59, 0.54)	-0.04 (-0.62, 0.54)	-0.41 (-1.27, 0.45)	-0.43 (-1.33, 0.47)
2 yr -4 yr	-0.49±3.38	Non- HDP	Reference	Reference	Reference	Reference	Reference	Reference
	-0.39±0.79	HDP	0.10 (-0.46, 0.66)	0.14 (-0.46, 0.74)	0.02 (-0.18, 0.23)	0.06 (-0.16, 0.28)	0.17 (-0.95, 1.30)	0.24 (-0.93, 1.42)
4yr- 7yr	0.26±22.6	Non- HDP	Reference	Reference	Reference	Reference	Reference	Reference
	0.22±1.76	HDP	-0.03 (-4.27, 4.20)	-0.02 (-4.61, 4.57)	-0.19 (-8.41, 8.02)	-0.05 (-0.92, 9.10)	0.12 (-0.68, 0.92)	0.04 (-0.79, 0.87)

BMI was measured in kg/m^2. *$p<0.05$, Adjusted covariates: Age at measurement, sex, mothers age, mothers pre pregnancy BMI, parity, smoking during first trimester, alcohol during first trimester. Sex was excluded from stratified analysis.
